# Mobile Assessments of Mood, Cognition, Smartphone-Based Sensor Activity, and Variability in Craving and Substance Use in Patients With Substance Use Disorders in Norway: Prospective Observational Feasibility Study

**DOI:** 10.2196/45254

**Published:** 2023-06-23

**Authors:** Anders Dahlen Forsmo Lauvsnes, Tor Ivar Hansen, Sebastian Øiungen Ankill, Sang Won Bae, Rolf W Gråwe, Taylor A Braund, Mark Larsen, Mette Langaas

**Affiliations:** 1 Department of Mental Health Faculty of Medicine and Health Sciences Norwegian University of Science and Technology Trondheim Norway; 2 Clinic of Substance Use and Addiction Medicine St Olavs University Hospital Trondheim Norway; 3 Department of Neuromedicine and Movement Science Faculty of Medicine and Health Sciences Norwegian University of Science and Technology Trondheim Norway; 4 Department of Mathematical Sciences Faculty of Information Technology and Electrical Engineering Norwegian University of Science and Technology Trondheim Norway; 5 Human-Computer Interaction and Human-Centered AI Systems Lab, AI for Healthcare Lab School of Systems and Enterprises Stevens Institute of Technology Hoboken, NJ United States; 6 Department of Research and Development Division of Psychiatry St Olavs University Hospital Trondheim Norway; 7 Black Dog Institute Sydney Australia; 8 Faculty of Medicine and Health University of New South Wales Sydney Australia

**Keywords:** executive functioning, substance use disorder, ecological momentary assessment, clinical inference, substance use, pilot study, mood, mental health, neurocognitive functioning, smartphone use, mobile sensor, sensor, decision support, mobile phone

## Abstract

**Background:**

Patients with substance use disorders (SUDs) are at increased risk for symptom deterioration following treatment, with up to 60% resuming substance use within the first year posttreatment. Substance use craving together with cognitive and mental health variables play important roles in the understanding of the trajectories from abstinence to substance use.

**Objective:**

This prospective observational feasibility study aims to improve our understanding of specific profiles of variables explaining SUD symptom deterioration, in particular, how individual variability in mental health, cognitive functioning, and smartphone use is associated with craving and substance use in a young adult clinical population.

**Methods:**

In this pilot study, 26 patients with SUDs were included at about 2 weeks prior to discharge from inpatient SUD treatment from 3 different treatment facilities in Norway. Patients underwent baseline neuropsychological and mental health assessments; they were equipped with smartwatches and they downloaded an app for mobile sensor data collection in their smartphones. Every 2 days for up to 8 weeks, the patients were administered mobile ecological momentary assessments (EMAs) to evaluate substance use, craving, mental health, cognition, and a mobile Go/NoGo performance task. Repeated EMAs as well as the smartphone’s battery use data were averaged across all days per individual and used as candidate input variables together with the baseline measures in models of craving intensity and the occurrence of any substance use episodes.

**Results:**

A total of 455 momentary assessments were completed out of a potential maximum of 728 assessments. Using EMA and baseline data as candidate input variables and craving and substance use as responses, model selection identified mean craving intensity as the most important predictor of having one or more substance use episodes and with variabilities in self-reported impulsivity, mental health, and battery use as significant explanatory variables of craving intensity.

**Conclusions:**

This prospective observational feasibility study adds novelty by collecting high-intensity data for a considerable period of time, including mental health data, mobile cognitive assessments, and mobile sensor data. Our study also contributes to our knowledge about a clinical population with the most severe SUD presentations in a vulnerable period during and after discharge from inpatient treatment. We confirmed the importance of variability in cognitive function and mood in explaining variability in craving and that smartphone usage may possibly add to this understanding. Further, we found that craving intensity is an important explanatory variable in understanding substance use episodes.

## Introduction

Patients with substance use disorders (SUDs) are at increased risk for symptom deterioration after periods of protracted abstinence or decreased consumption. Symptom deterioration includes increased craving, mental health, and somatic health problems. Resumed or increased substance use after treatment initiation occurs in as many as 60% of the patients in the year following treatment [1]. This change in substance use behavior is typically also associated with craving. Definitions of craving have some heterogeneity across time and publications [2]. Nevertheless, a common denominator is generally agreed to be varying degrees of a subjective desire to use psychoactive substances [3]. The relationship between substance use craving and use is complex [3] and probably presents differently in various stages of rehabilitation and abstinence [4].

Cognitive factors play an important role with respect to both craving and substance use behaviors. These factors play a central role in the understanding of the baseline probability of initiating substance use, later development of use patterns, clinical course, prognosis, and ultimately, treatment response and discontinuation [5]. Recent studies also indicate that cognitive functioning has significant variability within patients over time [6]. However, there is conflicting evidence as to whether cognitive functioning moderates the relationship between craving and substance use or is a predictor of either one in itself [7,8]. What is clear is that cognitive dysfunctions impact decision-making processes underlying substance use behaviors [9]. Hence, a better understanding of how state and trait cognitive functioning and mental health are associated with real-life substance use craving and use may help inform treatment and risk management, possibly creating a pathway to more personalized treatment options in patients with SUD [10].

There are considerable differences in how mental health, cognition, and substance use symptoms have been measured and operationalized in prospective studies of clinical populations with SUD. The most common involves using a comprehensive baseline measure to predict later relapse [11]. More recent studies have leveraged brief high-frequency ecological momentary assessment (EMA) data, which capture individual within-day changes [12], while many studies use a combination of these. When it comes to the assessment of cognitive functions especially, the traditional comprehensive laboratory-based neuropsychological testing procedures are being supplemented with inventory-based assessments [13] and ambulatory assessments using performance-based testing on smartphones [14,15]. Such ambulatory approaches have been successfully tested to improve the ecological validity and the understanding of the dynamics of cognitive processes in normal populations [16], psychiatric populations [15,17], and populations with SUDs [14]. Although sometimes used interchangeably, performance-based neuropsychological tasks and self-report of the same are probably complementary sources of information rather than overlapping measures of the same construct [18].

To optimize assessments of behavioral, mental, and cognitive states both in and out of the laboratory, work has gone into developing unobtrusive ways of collecting data [19] by using mobile sensing. It entails the real-time collection of data from a range of mobile and wearable sensors typically found in smartwatches and phones and investigating how they relate to various mental, cognitive, and health variables [16]. As for mental health variables, mobility, activity, and phone use have proved helpful in predicting the level of symptomatology [20,21]. In the more specific case of substance use research, mobile sensing has also been used successfully to detect actual intoxication [22]. However, this approach has been used less to study the associated clinical features and courses of craving, mental health, and cognition. Mobile sensing data are most often used in conjunction with some level of self-report in the form of EMA [22] and have enabled a new focus on analyzing the role of intraindividual variability in individuals with SUD symptoms. It should be noted that current research in this field has mainly been performed on community samples and has only, to a limited extent, been carried out with patients with more severe presentations of SUDs, leaving a void in the literature.

The main aim of this feasibility study was to investigate how individual variability in mental health, cognitive functioning, and smartphone use is associated with craving and substance use in an inpatient clinical population of young adults with SUDs in Norway. We aimed to analyze both baseline and EMAs of cognitive functioning and mental health self-reports to understand the ability of baseline and repeated measures data to explain the variability in craving and presence of substance use episodes.

## Methods

### Study Participants

#### Inclusion and Exclusion Criteria

Patients were included from 3 participating specialized substance use treatment institutions. The institutions differ in duration and urgency of admittance to treatment, ranging from acute detoxification to planned up to 12-month-long therapeutic community treatment. To be eligible for inclusion, the patients had to be admitted for at least 4 weeks, aged between 18 and 30 years at the time of inclusion, and able to communicate in a Scandinavian language. Acute suicidality and ongoing psychosis were the exclusion criteria.

#### Procedure

Local staff identified patients based on the inclusion and exclusion criteria and asked if they would be open to participation. They were then approached for informed consent and later inclusion assessments by the first author, a consultant psychologist with a specialty in addiction psychology, about 2 weeks prior to planned discharge. Immediately after informed consent was obtained, the patients went through baseline assessments and were equipped with a study smartwatch (Withings Move ECG), and they downloaded and started data collection with the Monsenso app [23]. No recharge of the smartwatches was required during the study period. Every 2 days, the patients received an SMS text message from an automated messaging service with an individual link to an EMA. The data from the phone and smartwatch were followed up daily by the first author who contacted the participants by phone to ensure compliance if data were not registered for 2 days. After 8 weeks, the first author met with the participants to check out from the study. Patients were compensated with 100 NOK (approximately US $10) per completed EMA for up to 28 sessions and were allowed to keep the smartwatch as an additional remuneration for their participation.

### Measurements

#### Baseline Assessments

##### Conners Continuous Performance Test-Third Edition

The Conners Continuous Performance Test-third edition (CPT-3) is a computer-based Go/NoGo assessment of state attention that measures aspects of inattention, impulsivity, and vigilance [24]. Previously, the CPT-2, which is very similar to the CPT-3, had demonstrated adequate internal consistency for commission errors (α=.90) and response time (α=.96) and is a valid state level measure of sustained attention and response inhibition [25].

##### Wechsler Abbreviated Scale of Intelligence

The Norwegian translation of the Wechsler Abbreviated Scale of Intelligence (WASI) was used to assess general intellectual abilities at baseline. This test contains 4 subtests and gives reliable estimates of both full-scale verbal and performance IQ, within less than 1 SD from the more elaborate WAIS-III (Wechsler Adult Intelligence Scale-third edition) estimates [26].

##### UPPS-P (Urgency, Premeditation, Perseverance, Sensation Seeking, and Positive Urgency) Scale

The Norwegian translation of the UPPS-P (urgency, premeditation, perseverance, sensation seeking, and positive urgency) short-form impulsive behavior scale is a 20-item questionnaire assessing different aspects of trait impulsivity. This form has previously been validated in populations with SUDs and has demonstrated adequate internal consistency as well as validity, with strong correlations and common factor structure with the full-form UPPS-P [27]. We used the second order factor of emotion-based rash action as a candidate explanatory variable in our modelling. This factor has been linked to substance use behavior in previous research [28].

##### Adult Attention-Deficit/Hyperactivity Disorder Self-Report Scale Screener

The variables of adult attention-deficit/hyperactivity disorder self-report scale-6 items (ASRS-6) screener were used as a proxy measure of self-reported cognitive dysfunction. The ASRS-6 has previously been validated in Norwegian and for populations with SUDs [29,30]. The ASRS-6 contains 2 subscales with adequate test-retest reliability of a measure of inattentiveness (ASRS inattentiveness, items 1-4, *r*=0.77) as well as hyperactivity/impulsivity (ASRS hyperactivity/impulsivity, items 5 and 6, *r*=0.70) [31]. We report average scores per item (range 0-4).

##### HADS (Hospital Anxiety and Depression Scale)

The HADS (Hospital Anxiety and Depression Scale) consists of 7 questions about depression and anxiety, with a range of 0-42 [32]. We did not employ any clinical cutoff scores in our analyses. This instrument has previously been validated in the Norwegian language, with adequate internal consistency, both for the depression (α=.76) and anxiety (α=.80) subscales [33] and has demonstrated adequate test-retest reliability [34]. We used the total score as a candidate explanatory variable.

#### Web-Based EMAs

The patients were sent an SMS text message every 48 hours with a link to a web-based EMA provided on the Memoro platform. The EMA had to be answered within 24 hours. We created variables for each patient with mean and standard deviation for each item across EMA sessions. The following items were part of the EMA.

##### Symptom Check List 5-Item Version

For repeated assessments of mental health distress, we used the Hopkins Symptom Check List 5-item version (SCL-5) that was administered every 48 hours. The psychometric validity of SCL-5 has been demonstrated in Norwegian samples, with acceptable internal consistency (α=.87) and external validity with high correlations with more elaborate instruments (SCL-10 × SCL-5, *r*=0.91) [35,36].

##### Substance Use and Craving

Substance intake during the last 48 hours was assessed as a self-reported binary variable (yes/no). A continuous visual analog scale ranging from 0 to 100 was used to assess craving in the last 48 hours. Single-item visual analog scales have previously been found to provide acceptable validity [3].

##### Complex Reaction Time

A complex reaction time (CRT) task was used to assess response inhibition and reaction time at each session every 48 hours. This test has a Go/NoGo paradigm consisting of 40 trials (interstimulus interval 1000 ms, 2000 ms, 4000 ms; 20% NoGo trials). Performance was measured as the number of valid responses (Go), correct inhibition of response to nontargets (correct-NoGo), omissions (failure to respond), and commissions (failure to inhibit response), in addition to the simple reaction time across all 40 trials (CRT-all reaction times) and standard deviation in reaction time across 40 trials (CRT-all standard deviations in reaction times). We used reaction time and commission errors as candidate explanatory variables. The mobile version of this test remains to be validated, but it has previously been validated in a tablet and PC format [37].

##### Impulsivity

At each EMA, patients were asked to rate their subjective impulsivity on items 12 and 17 from the Barrat Impulsiveness Scale (Norwegian version). These 2 items had the highest loading on cognitive and behavioral factors in the widely accepted 2-factor solution for the Barrat Impulsiveness Scale-11 in the Norwegian validation [38]. We calculated a composite score as the average of these 2 on each occasion, with item 12 being inversely coded, and referred to this candidate explanatory variable as impulsivity in this paper.

##### Mobile Sensing

The available smartphone and smartwatch usage and activity sensors included smartphone use sensors, accelerometer, and GPS. A full overview of the sensors and features is available on request. We wanted to use easily available sensor features to possibly allow for intuitive interpretations of results and easier implementation on other sensing platforms. For this feasibility study, to gain insights into the general phone usage patterns of our participants, we used data from the battery sensor to determine daily battery use (battery percentage points used during a day) as a proxy of daily smartphone usage (eg, app usage, communications) and created mean and standard deviation of battery use across all sessions.

### Statistical Analyses

#### Software

For all statistical analyses, the R statistical programming software was used [39].

#### Data Preparation

Mobile sensing data were provided by Monsenso in various epochs, triggered by activity and with a timestamp, enabling mobile sensing data to be summarized into 2-day epochs. EMA data were collected every second day for the whole study period (up to day 56). For each patient, we aggregated the scores across all available observations and created patient-wise mean (SD) for each EMA variable. These variables were then used as candidate explanatory variables for regression analyses. The mean of craving intensity was also used as a response. We further excluded all sessions directly preceded by substance use (self-reported substance use in previous 2 days). Then, to permit a cooldown period of up to 2 days, if the next session was not preceded by substance use during its previous 2 days, it was included again. This was to avoid data being directly influenced by the acute effects of substance use. Two patients were removed from modeling due to insufficient repeated data. One item from the UPPS-P first order factor “lack of premeditation” was missing in the Norwegian translation (item 19) for all patients. We calculated this factor as the mean of the 3 other items. This factor was, however, not part of the further analyses. To choose candidate explanatory variables, we started by studying the correlations between all repeated and baseline explanatory variables. We then removed strongly correlated variables within each measurement group (see sections 1 and 2 of the Multimedia Appendix 1 for details).

For missing data, we used mean imputation embedded in the mice package [40], resulting in the replacement of 4 missing data points at baseline and 1 for aggregated repeated measures. To check the robustness of the results from the statistical analysis of the mean imputed data, we also performed multiple imputation, which gave similar results as the results to be reported for the mean imputed data. The results from the multiple imputation and the available number of repeated data points per variable are available on request.

#### Descriptive Statistics

The median (IQR) across baseline, all sessions, and patients were used to describe input and response variables in our data set. Furthermore, the Wilcoxon rank-sum test was used to compare 2 distributions and Pearson product-moment correlations to investigate associations at the group level. For repeated measures, we also used repeated measures correlations between pairs of craving and the explanatory variables.

**Figure 1:**
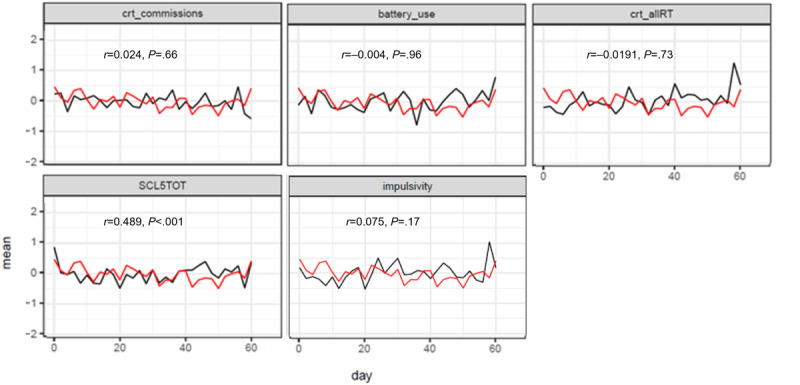
Daily display (x-axis) of craving intensity (red, y-axis) and the repeated measures (black, y-axis) later to be used as candidate explanatory variables (aggregated versions) in the stepwise statistical model selection. For the repeated measures, the mean is calculated by study day and across patients after repeated measures were centered and scaled for each patient. Repeated measures correlations between craving and the variable in question are given together with the associated *P* value. These data exclude sessions preceded by substance use. crt_allRT: Complex reaction Time: Reaction time; crt_commissions: Complex reaction Time: Commission errors; SCL5TOT: Symptom Check List 5-item version, total score.

The scale function in R was used to normalize the data to facilitate easy comparison of repeated measures data with different scales. This function employs mean (SD) over the entire data range (all sessions) for each individual, and the value represents the number of standard deviations from the individual mean. We then visualized the relationship between the repeated measures input data and craving by using the ggplot-function in the ggplot2 package in R [41] as shown in Figure 1 and examined the repeated measures correlations for these relationships by using the rmcorr function in R [42]. For all further statistical analyses, the unscaled raw data were used. We used the entire unimputed data set (N=26) for descriptive statistics at baseline. For repeated measures data, we removed 97 sessions preceded by substance use.

#### Statistical Modeling

As responses, we used the mean craving (across all sessions) for each patient and an indicator variable signifying if the patient had at least one episode of substance use during the study (referred to as substance use episodes). For the mean craving response, we fitted a multiple linear regression, and for the binary outcome of at least one substance use episode, we fitted a logistic regression. We found the best model fit for craving and binary substance use episodes by using automated stepwise model selection with the step Akaike information criterion function implemented in the MASS package in R [43]. A lower relative Akaike information criterion indicates a better fit with less complexity, and models with the lowest Akaike information criterion were thus preferred. For final models, we estimated the variation inflation factor to diagnose any remaining issues with multicollinearity and suppression effects. *P* values were calculated from the *t* distribution for the linear regression model and by using the normal approximation for the logistic regression models. The amount of variance explained by the models was estimated by *R*^2^ and McFadden *R*^2^ for linear and logistic regression models, respectively.

### Ethics Approval

This study was conducted according to the declaration of Helsinki and approved by the Regional Committee for Medical and Health Research Ethics Southeast Norway (approval 269117) on October 1, 2021. Informed consent was obtained from the participants upon the start of the data collection. Data were collected and stored according to the appropriate national and European legislation governing data privacy in research on an encrypted secure server. This was preapproved by the Regional Committee for Medical and Health Research Ethics Southeast Norway.

## Results

In this study, 26 patients (7 females, 27%) were included, and they contributed to 455 (62.5% compliance) EMA sessions out of a maximum possible of 728 assessments; this is slightly lower than the average compliance in clinical populations with SUDs [44]. We aimed to include the patients 14 days before their planned discharge (mean 14.4 [SD 10.1] days, range 1-43 days). Fourteen patients reported substance use preceding 97 EMA reports (mean 6.92, range 0-17). Descriptive statistics and between-group analyses for patients with and or without substance use episodes of candidate explanatory variables are shown in Table 1.

Table 1 summarizes all the candidate explanatory variables and their descriptive properties. Patients with and without substance use episodes did not present with different general ability (IQ) levels (WASI scores). It should be noted that the span of IQ scores was broad, including patients with borderline intellectual disabilities. Patients with substance use episodes had significantly more baseline commission errors on the CPT (*P*=.048); they had a higher mean of repeated impulsivity scores (*P*=.046). Further, patients with substance use episodes had a higher mean of craving intensity (*P*=.004) than patients without substance use episodes. Table 2 gives an outline of the substance use patterns of the patients.

Figure 1 shows the covariability of craving intensity and the 5 repeated measures collected from the EMA. SCL-5 total score had the only significant repeated measure correlation (*r*=0.489; *P*<.001) with craving intensity. All fitted regression models are summarized in Table 3.

**Table 1:** Baseline group level descriptive statistics and between-group comparisons for patients with and without substance use episodes during the study period for all candidate explanatory variables.

				Full sample (N=26), median (IQR)		Patients with substance use episode(s) (n=14), median (IQR)		Patients with no substance use episode(s) (n=12), median (IQR)		*P* value
Age (years)				26.37 (4.18)		25.57 (3.60)		27.00 (4.45)		.67
**Baseline measures**										
	**Wechsler Abbreviated Scale of Intelligence**									
		Full-scale IQ estimate	89.00 (17.50)		87.50 (12.00)		89.00 (12.00)		.88	
	**Conners Continuous Performance Test-third edition**									
		Commissions	64.00 (15.00)		68.50 (13.75)		59.00 (11.00)		.048	
		Reaction time consistency (SD of hit reaction time)	49.00 (13.00)		50.50 (11.50)		48.00 (12.00)		.62	
	**Adult Attention-Deficit/Hyperactivity Disorder Self-Report Scale screener**									
		Factor 1: inattentiveness	2.50 (1.25)		2.75 (1.25)		2.13 (1.06)		.07	
		Factor 2: hyperactivity/impulsivity	3.00 (1.00)		3.00 (1.00)		2.75 (0.88)		.34	
	**HADS (Hospital Anxiety and Depression Scale)**									
		Total	18.00 (9.75)		17.50 (11.50)		19.00 (5.25)		.92	
	**UPPS-P (urgency, premeditation, perseverance, sensation seeking, and positive urgency)**									
		Emotion-Based Rash Action	2.71 (0.42)		2.73 (0.34)		2.71 (0.34)		.87	
**Repeated measures**										
	**Complex reaction time (mean across sessions)**									
		Reaction time (ms)	483.79 (77.82)		483.80 (54.04)		480.29 (12.84)		.77	
		Commission errors	4.93 (5.31)		4.87 (2.49)		6.00 (13.21)		>.99	
	Impulsivity mean			4.78 (1.58)		5.16 (1.076)		4.00 (1.264)		.046
	Symptom Check List 5-item version			9.63 (4.40)		10.05 (4.40)		9.59 (4.01)		.60
	Craving (0-100)			33.88 (37.01)		45.65 (25.42)		22.00 (15.78)		.004
	Mobile sensors: battery use (battery percentage points used during a day, mean across sessions)			62.18 (41.39)		58.18 (49.30)		62.18 (18.69)		.54

**Table 2:** Substance use patterns of the patients (N=26).

Substance	Primary substance (n)	Secondary substance (n)
Alcohol	5	6
Opiates	3	1
Cannabinoids	7	5
Sedatives, hypnotics	3	8
Cocaine	2	2
Stimulants other than cocaine	5	7
Hallucinogens	1	5

**Table 3:** Summary of the results from final regression models with craving intensity (linear regression) and substance use episodes (logistic regression) as response.^a^

Model				Estimate		Odds ratio (95% CI)		SE		*R* ^2^			*P* value	
**Baseline models**														
	**Response: Craving (mean)**										.254			N/A^b^
		Intercept	–18.56		N/A		22.79		N/A			.43		
		CPT^c^ reaction time consistency (SD of hit reaction time)	0.60		N/A		0.37		N/A			.12		
		ASRS^d^ inattentiveness	9.99		N/A		5.46		N/A			.08		
	**Response: Substance use episodes (binary)**										.186			N/A
		Intercept	–4.98		0.01 (0.00-0.97)		0.04		N/A			.08		
		CPT commissions	0.08		1.09 (1.004-1.20)		0.04		N/A			.06		
**Repeated measures models**														
	**Response: Craving (mean)**										.680			N/A
		Intercept	26.03		N/A		28.89		N/A			.38		
		SCL-5^e^ (mean)	3.98		N/A		1.13		N/A			.003		
		Impulsivity (mean)	7.26		N/A		2.57		N/A			.01		
		Battery use (mean)	–0.23		N/A		0.10		N/A			.04		
		CRT^f^ all reaction times (mean)	–0.09		N/A		0.05		N/A			.09		
		CRT commissions (mean)	–0.50		N/A		0.31		N/A			.13		
	**Response: Substance use episodes (binary)**										.366			N/A
		Intercept	0.15		1.17 (0.02-76.57)		1.97		N/A			.94		
		Craving (mean)	0.12		1.13 (1.04-1.30)		0.05		N/A			.02		
		SCL-5 (mean)	–0.43		0.65 (0.30-1.09)		0.31		N/A			.17		

^a^The estimated regression coefficients are denoted as estimate, and estimated standard errors of the regression coefficient are denoted as SE. The *R*^2^ gives the proportion of explained variability for the linear regression and the McFadden *R*^2^ for the logistic regression model. The *P* value is for the test wherein the regression coefficient is equal to 0.

^b^N/A: not applicable.

^c^CPT: Conners Continuous Performance Test.

^d^ASRS: adult attention-deficit/hyperactivity disorder self-report scale.

^e^SCL-5: Symptom Check List 5-item version.

^f^CRT: complex reaction time.

For mean craving intensity and substance use episodes as responses, there were no significant baseline explanatory variables. Using aggregated repeated measures data (across sessions) as input, we found that impulsivity (impulsivity mean estimate 7.26, SE 2.57; *P*=.01), mood (SCL-5 mean estimate 3.98, SE 1.13; *P*=.003), and battery use (battery use mean estimate –0.23, SE 0.10; *P*=.04) were the significant explanatory variables of craving intensity. For substance use episodes as response, the best model fit had mean craving intensity (craving mean estimate 0.12, SE 0.05, odds ratio 1.13, 95% CI 1.04-1.30; *P*=.04) as the sole significant explanatory variable, albeit with modest effect size. Both models using repeated measures data explained more variability in substance use episodes and craving than the baseline models (see Table 3 for *R*^2^).

## Discussion

### Principal Findings

The main aim of this study was to investigate how mental health and cognitive functioning measured at both baseline and with frequent EMAs in addition to smartphone-based sensor activity is associated with variability in craving and actual substance use in a clinical population of young adults aged 18 to 30 years. To the best of our knowledge, we are the first to demonstrate these relationships with high frequency data collection in an inpatient clinical population of young adults with SUDs. Overall, our results indicate that repeated measures outperform baseline measures in explaining variability in craving (*R*^2^=0.680 vs 0.254, respectively) and substance use episodes (*R*^2^=0.366 vs 0.186, respectively). Using a cross-sectional breakdown of the data, with repeated measures aggregated by patient in addition to baseline mental health and neuropsychological performance data, we found that having one or more episodes of substance use in the study period was best explained by mean craving intensity. We also found that repeated mental health assessments, self-reported impulsivity, as well as the digital marker of amount of smartphone usage (battery use) are possible explanatory variables of substance use episodes through their effect on craving. Mean craving intensity was best explained by repeated mental health assessments and self-reported impulsivity. Less smartphone-based sensor activity (ie, battery use) was also a significant explanatory variable, albeit with a modest effect. This means that increases in mental health distress and perceived impulsivity will increase the level of craving intensity, while increases in the amount of phone use, as measured by battery use, will decrease craving intensity. Interestingly, we were able to find these differences even in a relatively small sample of only clinical patients with SUDs and not in patients with SUDs versus healthy controls in this feasibility study. There has been some discussion in the literature about whether self-regulatory mechanisms such as cognitive functions and disinhibition influence the vulnerability to act on craving and use substances [45], but we did not find support for this in our study, as no measures of cognitive functioning were significant in explaining variability in substance use episodes; we did, however, not allow for an interaction term.

### Comparison With Previous Work

Our results are in line with other studies documenting the relationship between craving and substance use [46], and the modest effect size that we found is in line with previous findings [3], but previous studies did not consistently account for within-subject effects such as individual levels of craving and variability across time. Similarly, since there was an important variability in baseline cognitive functioning, it may be that models allowing for random intercepts and random slopes in a nested mixed-effects model would have nuanced the relationship among craving, cognitive factors, and substance use episodes. This trade-off was made due to better possibilities for variable selection and handling of a high number of possible explanatory variables in an aggregated approach such as the one used here.

The influence of mood and impulsivity on substance use has previously been described for patients with various severities of addiction [5,47]. We did not, however, perform analyses of battery use as a possible explanatory variable for variability in mood and impulsivity, but battery use was significantly correlated with the mean of repeated mood assessments and could have proven to be an explanatory variable of variability in mood. These findings add to our understanding of the importance of incorporating dynamic measures in clinical risk assessments and future studies, as has also been suggested by others [48], and incorporating digital markers such as smartphone use, as they may provide even earlier signals of impending substance use.

Emotion-based rash action was not part of any of the final baseline models nor was any baseline neuropsychological measure; this is in contrast with what has been reported in some previous studies [49,50], as it seems that in our study, self-reported cognitive dysfunction, as measured by ASRS, is better at explaining variability in craving intensity from baseline than performance measures and trait-based measures, albeit not significantly in our analyses. This finding further supports the notion that self-report and performance-based cognitive measures may play different roles in explaining variability in outcomes such as substance use outcomes, as do time varying and baseline explanatory variables [5].

### Further Research

A review [51] concluded that increasing the frequency of measures and analyzing several sessions simultaneously rather than separately may improve task reliability and validity in populations with SUDs and that analyzing several sessions at once, using variability for different epochs of time, rather than just session by session, may improve both the reliability of measures and the validity of findings in this population. Further studies should explore features of different epochs of data, especially from neuropsychological data.

In this study, we aggregated average repeated measures data across sessions and patients, which may not be optimal in cases where there are substantial individual differences [52]. The effects of large variability in cognitive functioning and mental distress between patients both at baseline and over time may be masked on an aggregated group level. As an alternative, repeated assessments may be nested within individuals and analyzed using a mixed-effects approach. This may improve the performance of these models by incorporating time dependency in observations. Further, mixed-effects modeling enables prospective analyses with lagged input for a better temporal variance structure. As an example, patients may express more impulsivity and substance use behaviors in general (accounted for in this study) but not necessarily within a certain time epoch (eg, one day, not accounted for in this study). These levels of analyses both between and within-patients are important to understand with respect to those who have an increased risk of substance use as well as when. The drawback with mixed-effects approaches is that they are not yet compatible with a large number of candidate explanatory variables and techniques for effective variable selection. The first step of candidate explanatory variable selection of the aggregated variables was empirically driven and is outlined in Multimedia Appendix 1. In the second step, we used stepwise Akaike information criterion regression for explanatory variable selection. There are other statistical methods available for variable selection, such as the lasso (and elastic net) regression. The analyses in this work were performed with both mean imputation (as presented in the main text) and verified with multiple imputation. We chose a variable selection method that could be combined with both mean and multiple imputation and would give both confidence intervals for regression coefficients and *P* values. Based on this feasibility study, we find it possible to aim for an expansion of the data set in the future. This kind of data collection is challenging both for patients and researchers; therefore, evaluating feasibility and limiting and tuning the extent and timing of data collection is of great importance for future studies.

Our sample size in this feasability study limits the generalizability of our findings and has made sublevel analyses based on biological sex, high/low craving, drug of choice, or cognitive subgroups difficult. Although we do consider our sample to be representative of the clinical cohort in this age bracket, some of these variables may have effect on the outcomes in this study. Future studies with larger sample sizes would allow further subgroup analyses based on these factors and other sociodemographic variables such as biological sex or educational background. For example, Serre et al [8] found differential relationships between craving and mood and substance use depending on substance of choice. Identifying such substance-specific mood and cognitive risk factors may contribute to improving the possibilities for targeted individualized interventions for this population.

Further, although we were able to differentiate patients with substance use episodes from those who did not have substance use episodes in this small clinical cohort, it could be beneficial to add control groups with and without substance use problems. Additionally, to personalize treatments, the identification of imminent risk and ongoing substance use is important. In our study, we removed sessions preceded by substance use because we wanted to study the risk of craving and use rather than substance use–influenced sessions themselves. It may, however, be argued that the acute effects of substance use are individual- and drug-specific and may exceed this cooldown period, possibly influencing our data and findings. Future research may thus want to use classification tools to enable phenotyping of the substance use episodes themselves. To enable accurate classification of ongoing substance use, the reliability of the substance use measure is crucial. In this study, we relied on self-report, which has indeed shown varying reliability, often decreasing with time in prospective studies. Employing biological markers in the form of randomized or fixed schedule sampling of urine or blood outside the clinic is one possibility that should be considered in future clinical and prospective research with this population. The downside to such, more reliable, but also more intrusive approaches is that they could have led to attrition and changes in drug use or self-reports.

### Conclusion

Repeated measures data of mental health, cognition, and smartphone usage play an important role in explaining the variability in mean craving intensity and the occurrence of substance use episodes for patients with severe SUDs. This feasibility study, albeit small in sample size, adds novelty and knowledge to substance use research when using intense data collection methods in a group of patients with high symptom load. Due to the small sample size of this feasibility study, we refrain from drawing strong conclusions based on the significance of the regression coefficients. We were also able to show that it is feasible, albeit work-demanding, to conduct this kind of intensive prospective study, including mobile neuropsychological performance measures, in this patient cohort, which represents one of the most severe clinical cohorts of patients with SUDs. This study has suggestions for further research and underlines the need to target craving, cognition, and mood in the personalized assessment and treatment planning in this population.

## Appendix

Multimedia Appendix 1 Correlation plots used for candidate explanatory variable selection.

## Abbreviations

ASRS-6: adult attention-deficit/hyperactivity disorder self-report scale-6 items
CPT-3: Conners Continuous Performance Test-third edition
CRT: complex reaction time
EMA: ecological momentary assessment
HADS: Hospital Anxiety and Depression Scale
SCL-5: Symptom Check List 5-item version
SUD: substance use disorder
UPPS-P: urgency, premeditation, perseverance, sensation seeking, and positive urgency
WAIS-III: Wechsler Adult Intelligence Scale-third edition
WASI: Wechsler Abbreviated Scale of Intelligence

## Data Availability

The data sets generated during and analyzed during this study are not publicly available due to the high risk of reidentification. The data may be made available based on a reasonable request accompanied by an approval from the Regional Committee for Medical and Health Research Ethics Southeast Norway.

## References

[1] Buckheit KA, Moskal D, Spinola S, Maisto SA (2018). Clinical Course and Relapse among Adolescents Presenting for Treatment of Substance Use Disorders: Recent Findings. Curr Addict Rep.

[2] Flaudias V, Heeren A, Brousse G, Maurage P (2019). Toward a Triadic Approach to Craving in Addictive Disorders: The Metacognitive Hub Model. Harv Rev Psychiatry.

[3] Cavicchioli M, Vassena G, Movalli M, Maffei C (2020). Is craving a risk factor for substance use among treatment-seeking individuals with alcohol and other drugs use disorders? A meta-analytic review. Drug Alcohol Depend.

[4] Moe F, Moltu C, McKay J, Nesvåg S, Bjornestad J (2022). Is the relapse concept in studies of substance use disorders a 'one size fits all' concept? A systematic review of relapse operationalisations. Drug Alcohol Rev.

[5] Vassileva J, Conrod PJ (2019). Impulsivities and addictions: a multidimensional integrative framework informing assessment and interventions for substance use disorders. Philos Trans R Soc Lond B Biol Sci.

[6] Konova AB, Lopez-Guzman S, Urmanche A, Ross S, Louie K, Rotrosen J, Glimcher PW (2020). Computational Markers of Risky Decision-making for Identification of Temporal Windows of Vulnerability to Opioid Use in a Real-world Clinical Setting. JAMA Psychiatry.

[7] Remmerswaal D, Jongerling J, Jansen P, Eielts C, Franken I (2019). Impaired subjective self-control in alcohol use: An ecological momentary assessment study. Drug Alcohol Depend.

[8] Serre F, Fatseas M, Denis C, Swendsen J, Auriacombe M (2018). Predictors of craving and substance use among patients with alcohol, tobacco, cannabis or opiate addictions: Commonalities and specificities across substances. Addict Behav.

[9] Ahn W, Ramesh D, Moeller FG, Vassileva J (2016). Utility of Machine-Learning Approaches to Identify Behavioral Markers for Substance Use Disorders: Impulsivity Dimensions as Predictors of Current Cocaine Dependence. Front Psychiatry.

[10] Verdejo-Garcia A, Garcia-Fernandez G, Dom G (2022). Cognition and addiction. Dialogues in Clinical Neuroscience.

[11] Lima D, Gonçalves PD, Ometto M, Malbergier A, Amaral R, Dos Santos B, Cavallet M, Chaim-Avancini T, Serpa MH, Ferreira LRK, Duran FLS, Zanetti MC, Nicastri S, Busatto GF, Andrade AG, Cunha PJ (2019). The role of neurocognitive functioning, substance use variables and the DSM-5 severity scale in cocaine relapse: A prospective study. Drug Alcohol Depend.

[12] Scott CK, Dennis ML, Gustafson DH (2018). Reprint of Using ecological momentary assessments to predict relapse after adult substance use treatment. Addict Behav.

[13] Hagen E, Erga A, Hagen K, Nesvåg SM, McKay J, Lundervold A, Walderhaug E (2016). Assessment of Executive Function in Patients With Substance Use Disorder: A Comparison of Inventory- and Performance-Based Assessment. J Subst Abuse Treat.

[14] Chung T, Bae SW, Mun E, Suffoletto B, Nishiyama Y, Jang S, Dey AK (2020). Mobile Assessment of Acute Effects of Marijuana on Cognitive Functioning in Young Adults: Observational Study. JMIR Mhealth Uhealth.

[15] Lavigne KM, Sauvé G, Raucher-Chéné D, Guimond S, Lecomte T, Bowie CR, Menon M, Lal S, Woodward TS, Bodnar MD, Lepage M (2022). Remote cognitive assessment in severe mental illness: a scoping review. Schizophrenia.

[16] Tseng VW, Costa JDR, Jung MF, Choudhury T (2020). Using Smartphone Sensor Data to Assess Inhibitory Control in the Wild: Longitudinal Study. JMIR Mhealth Uhealth.

[17] Cormack F, McCue M, Taptiklis N, Skirrow C, Glazer E, Panagopoulos E, van Schaik TA, Fehnert B, King J, Barnett JH (2019). Wearable Technology for High-Frequency Cognitive and Mood Assessment in Major Depressive Disorder: Longitudinal Observational Study. JMIR Ment Health.

[18] Snyder HR, Friedman NP, Hankin BL (2021). Associations Between Task Performance and Self-Report Measures of Cognitive Control: Shared Versus Distinct Abilities. Assessment.

[19] Wen H, Sobolev M, Vitale R, Kizer J, Pollak JP, Muench F, Estrin D (2021). mPulse Mobile Sensing Model for Passive Detection of Impulsive Behavior: Exploratory Prediction Study. JMIR Ment Health.

[20] Currey D, Torous J (2022). Digital phenotyping correlations in larger mental health samples: analysis and replication. BJPsych Open.

[21] Zhang Y, Folarin AA, Sun S, Cummins N, Vairavan S, Bendayan R, Ranjan Y, Rashid Z, Conde P, Stewart C, Laiou P, Sankesara H, Matcham F, White KM, Oetzmann C, Ivan A, Lamers F, Siddi S, Vilella E, Simblett S, Rintala A, Bruce S, Mohr DC, Myin-Germeys I, Wykes T, Haro JM, Penninx BW, Narayan VA, Annas P, Hotopf M, Dobson RJ, RADAR-CNS consortium (2022). Longitudinal Relationships Between Depressive Symptom Severity and Phone-Measured Mobility: Dynamic Structural Equation Modeling Study. JMIR Ment Health.

[22] Bae S, Chung T, Islam R, Suffoletto B, Du J, Jang S, Nishiyama Y, Mulukutla R, Dey A (2021). Mobile phone sensor-based detection of subjective cannabis intoxication in young adults: A feasibility study in real-world settings. Drug Alcohol Depend.

[23] Monsenso.

[24] Scimeca LM, Holbrook L, Rhoads T, Cerny BM, Jennette KJ, Resch ZJ, Obolsky MA, Ovsiew GP, Soble JR (2021). Examining Conners Continuous Performance Test-3 (CPT-3) Embedded Performance Validity Indicators in an Adult Clinical Sample Referred for ADHD Evaluation. Dev Neuropsychol.

[25] Shaked D, Faulkner LMD, Tolle K, Wendell CR, Waldstein SR, Spencer RJ (2020). Reliability and validity of the Conners' Continuous Performance Test. Appl Neuropsychol Adult.

[26] Bosnes O (2009). Norsk versjon av Wechsler Abbreviated Scale of Intelligence: Hvor godt er samsvaret mellom WASI og norsk versjon av Wechsler Adult Intelligence Scale-III?. Tidsskrift for Norsk Psykologforening.

[27] Cyders MA, Littlefield AK, Coffey S, Karyadi KA (2014). Examination of a short English version of the UPPS-P Impulsive Behavior Scale. Addict Behav.

[28] Smith GT, Cyders MA (2016). Integrating affect and impulsivity: The role of positive and negative urgency in substance use risk. Drug Alcohol Depend.

[29] Bu E, Skutle A, Dahl T, Løvaas E, van de Glind G (2012). Validering av ADHD-screeninginstrumentet ASRS-v1 for pasienter i behandling for rusmiddelavhengighet. Tidsskrift for norsk psykologforening.

[30] Kornør H, Hysing M (2011). Måleegenskaper ved den norske versjonen av Adult ADHD Self Report Scale, 1.1 (ASRS). PsykTestBarn.

[31] Hesse M (2013). The ASRS-6 has two latent factors: attention deficit and hyperactivity. J Atten Disord.

[32] Zigmond AS, Snaith RP (1983). The hospital anxiety and depression scale. Acta Psychiatr Scand.

[33] Mykletun A, Stordal E, Dahl AA (2001). Hospital Anxiety and Depression (HAD) scale: factor structure, item analyses and internal consistency in a large population. Br J Psychiatry.

[34] White D, Leach C, Sims R, Atkinson M, Cottrell D (1999). Validation of the Hospital Anxiety and Depression Scale for use with adolescents. Br J Psychiatry.

[35] Strand BH, Dalgard OS, Tambs K, Rognerud M (2003). Measuring the mental health status of the Norwegian population: a comparison of the instruments SCL-25, SCL-10, SCL-5 and MHI-5 (SF-36). Nord J Psychiatry.

[36] Tambs K, Moum T (1993). How well can a few questionnaire items indicate anxiety and depression?. Acta Psychiatr Scand.

[37] Hansen T, Lehn H, Evensmoen H, Håberg A (2016). Initial assessment of reliability of a self-administered web-based neuropsychological test battery. Computers in Human Behavior.

[38] Lindstrøm JC, Wyller NG, Halvorsen MM, Hartberg S, Lundqvist C (2017). Psychometric properties of a Norwegian adaption of the Barratt Impulsiveness Scale-11 in a sample of Parkinson patients, headache patients, and controls. Brain Behav.

[39] R Core Team (2022). R: A language and environment for statistical computing. R-Project.

[40] van Buuren S, Groothuis-Oudshoorn K (2011). mice: Multivariate Imputation by Chained Equations in R. J. Stat. Soft.

[41] Wickham H (2016). ggplot2: Elegant Graphics for Data Analysis.

[42] Bakdash J, Marusich L rmcorr: repeated measures correlation. CRAN.R-project.

[43] Venables WN, Ripley BD (2002). Modern Applied Statistics with S. 4th ed.

[44] Jones A, Remmerswaal D, Verveer I, Robinson E, Franken IHA, Wen CKF, Field M (2019). Compliance with ecological momentary assessment protocols in substance users: a meta-analysis. Addiction.

[45] Jakubiec L, Chirokoff V, Abdallah M, Sanz-Arigita E, Dupuy M, Swendsen J, Berthoz S, Gierski F, Guionnet S, Misdrahi D, Serre F, Auriacombe M, Fatseas M (2022). The Executive Functioning Paradox in Substance Use Disorders. Biomedicines.

[46] Vafaie N, Kober H (2022). Association of Drug Cues and Craving With Drug Use and Relapse: A Systematic Review and Meta-analysis. JAMA Psychiatry.

[47] Fatseas M, Serre F, Swendsen J, Auriacombe M (2018). Effects of anxiety and mood disorders on craving and substance use among patients with substance use disorder: An ecological momentary assessment study. Drug Alcohol Depend.

[48] Verdejo-Garcia A, Albein-Urios N (2021). Impulsivity traits and neurocognitive mechanisms conferring vulnerability to substance use disorders. Neuropharmacology.

[49] Stautz K, Cooper A (2013). Impulsivity-related personality traits and adolescent alcohol use: a meta-analytic review. Clin Psychol Rev.

[50] VanderVeen J, Hershberger A, Cyders M (2016). UPPS-P model impulsivity and marijuana use behaviors in adolescents: A meta-analysis. Drug Alcohol Depend.

[51] Zech HG, Reichert M, Ebner-Priemer UW, Tost H, Rapp MA, Heinz A, Dolan RJ, Smolka MN, Deserno L (2022). Mobile Data Collection of Cognitive-Behavioral Tasks in Substance Use Disorders: Where Are We Now?. Neuropsychobiology.

[52] Bakdash JZ, Marusich LR (2017). Repeated Measures Correlation. Front Psychol.

