# Supplementary material for: Mobile Assessments of Mood, Cognition, Smartphone-Based Sensor Activity, and Variability in Craving and Substance Use in Patients With Substance Use Disorders in Norway: Prospective Observational Feasibility Study
Source: JMIR Form Res. 2023 Jun 23;7:e45254. doi: 10.2196/45254 (PMC10337471; doi:10.2196/45254)
Supplement: Multimedia Appendix 1 [file formative_v7i1e45254_app1.pdf]

Appendix 1: Correlation plots used for candidate explanatory variable selection

"Mobile Assessments of Mood, Cognition, Smartphone-Based Sensor Activity, and Variability in Craving and Substance Use in Patients With Substance Use Disorders in Norway: Prospective Observational Feasibility Study"

Authors: Anders Dahlen Forsmo Lauvsnes, Tor Ivar Hansen, Sebastian Øiungen Ankill, Sang Won Bae, Rolf W. Gråwe, Taylor A. Braund, Mark E. Larsen & Mette Langaas

Including all baseline or repeated measures aggregated variables in our regression analyses is not possible due to our small sample size and large number of highly correlated covariates, since this would lead poor estimation of effects. In this appendix we show how the candidate explanatory variables were selected based on studying correlation plots. These candidate explanatory variables were then the starting point for the stepwise AIC model selection, as reported in the main article.

Section 1: Baseline covariates (14 variables)

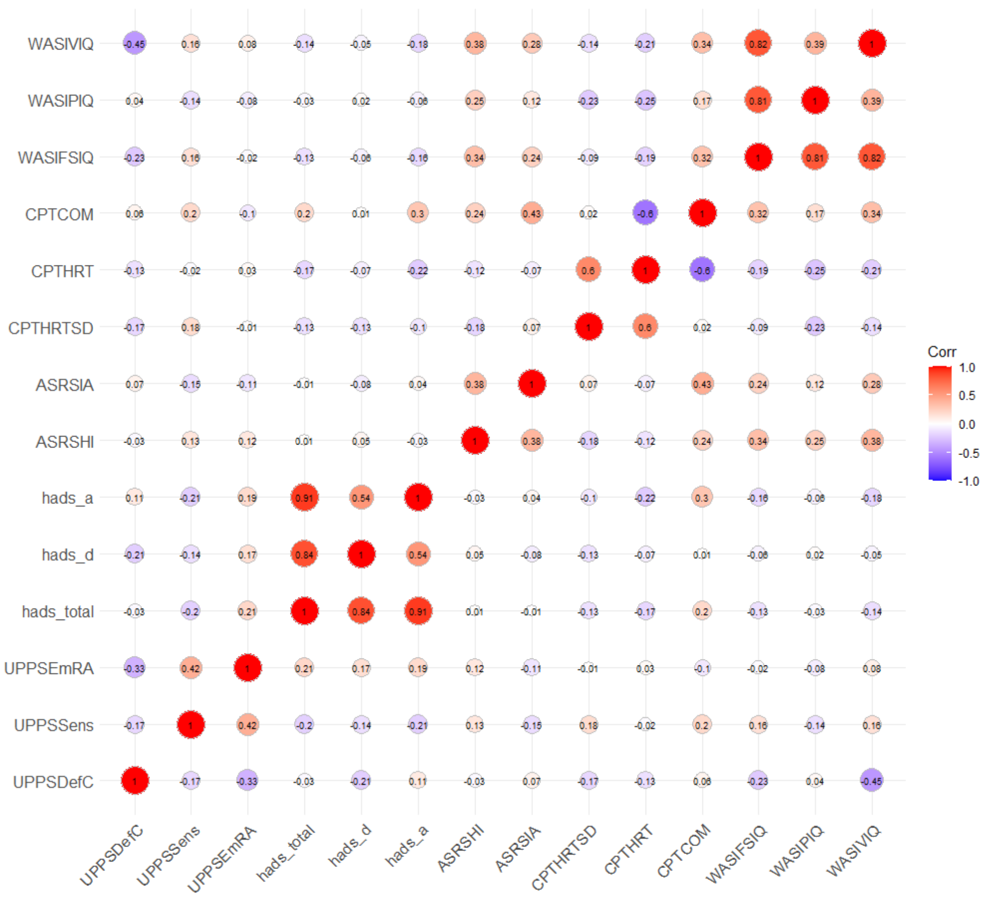

Abbreviations used and conclusions from studying the correlation plot at baseline

Wechsler abbreviated scale of intelligence (3 variables)

- WASIVIQ: Wechsler abbreviated scale of intelligence, verbal scale IQ-equivalents
- WASIPIQ: Wechsler abbreviated scale of intelligence, performance scale IQ-equivalents
- WASIFSIQ: Wechsler abbreviated scale of intelligence, full scale IQ estimate

Only *WASIFSIQ* (referred to as Wechsler Abbreviated Scale of Intelligence, Full Scale IQ estimate, in Table 1 of the main article) is included as a candidate explanatory variable.

CPT: Continuous Performance Test (3 variables)

- CPTCOM: CPT Commission errors
- CPTHRT: CPT Hit Reaction Time
- CPTHRTSD: CPT Hit Reaction Time SD

Both *CPTCOM* (referred to as Conners Continuous Performance Test-third edition, Commissions in Table 1 of the main article) and *CPTHRTSD* (referred to as Reaction time consistency SD of hit reaction time in Table 1 of the main article) are included as candidate explanatory variables.

ASRS: Adult ADHD Self-Report Scale (2 variables)

- ASRSIA: ASRS Inattentiveness
- ASRSHI: ASRS Hyperactivity/Impulsivity

Both variables *ASRSIA* and *ASRSHI* are included as candidate explanatory variables (referred to as Adult Attention-Deficit/Hyperactivity Disorder Self-Report Scale screener, Factor 1 and Factor 2, in Table 1 of the main article).

HADS: Hospital Anxiety and Depression Scale (3 variables)

- HADS_A: HADS Anxiety
- HADS_D: HADS Depression
- HADS_TOTAL: HADS Total score

Only *hads_total* (referred to as Hospital Anxiety and Depression Scale, Total, in Table 1 of the main article) is included as a candidate explanatory variable.

UPPS-P Short version (3 variables)

- UPPSEmRA: UPPS Emotion Based Rasch Action
- UPPSSens: UPPS Sensation Seeking
- UPPSDefC: UPPS Deficit in Conscientiousness

UPPSEmRA (referred to as UPPS-P (urgency, premeditation, perseverance, sensation seeking, and positive urgency) Emotion-Based Rash Action in Table 1 of the main article) is included as a candidate explanatory variable.

In total 7 candidate explanatory variables are chosen. The strongest correlation within this set is between ASRS AI and CPTCOM, with value 0.43.

Section 2: Repeated measures covariates (12 variables)

Remark: In our regression models either craving mean or binary substance use episodes is used as response. The craving mean is also included as an explanatory variable for the binary substance use episodes. The craving mean (repeated) measure is not included in the correlation plot below and is a candidate explanatory variable *in addition* to the variables identified below.

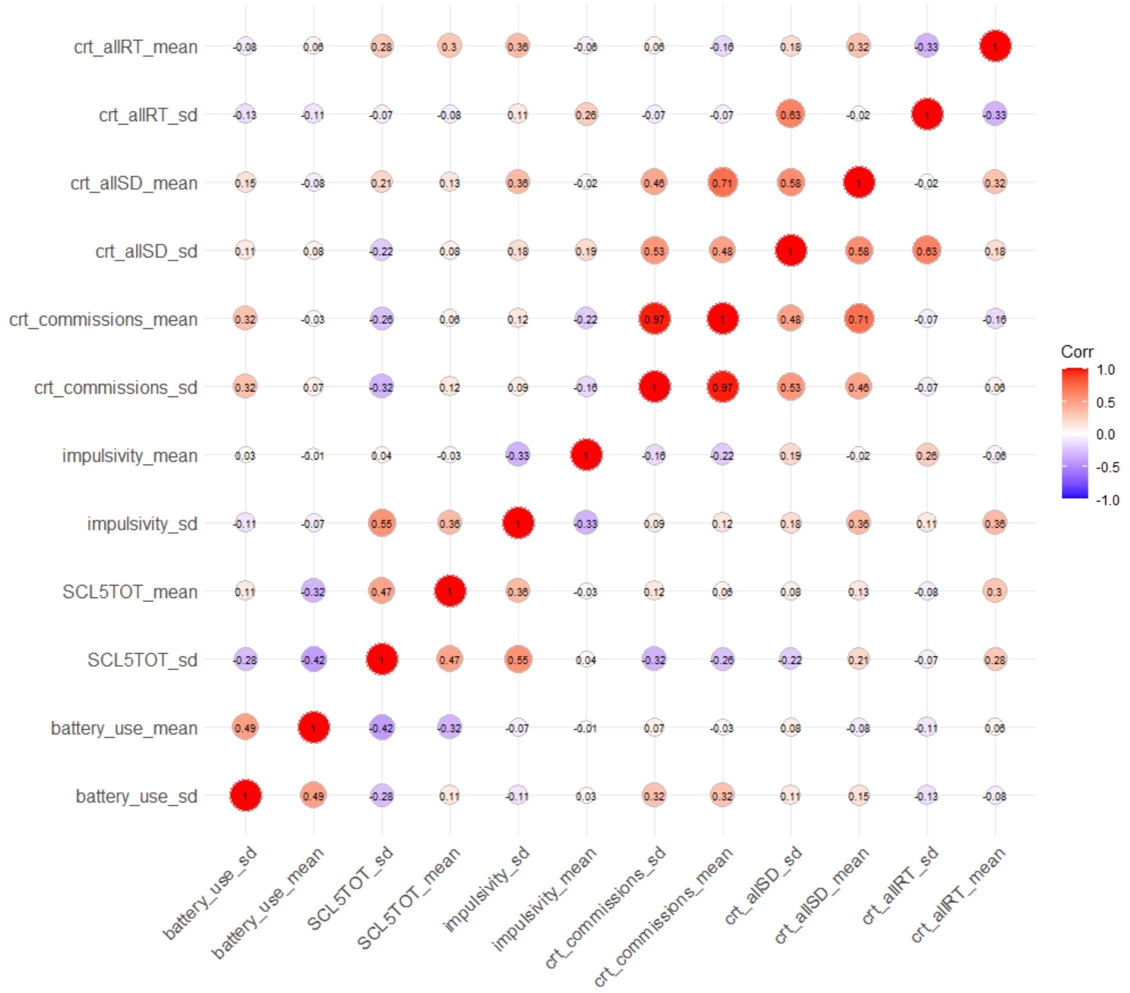

Abbreviations used in the used and conclusions from studying the correlation plot of repeated measures

Complex reaction time variables (6 variables):

- crt_allRT_mean: Mean of mean CRT Reaction time across trials (40 trials per session)
- crt_allRT_sd: Standard deviation of mean CRT Reaction time across trials (40 trials per session)
- crt_allSD_mean: Mean of standard deviation of CRT Reaction time across trials (40 trials per session)
- crt_allSD_sd: Standard deviation of the standard deviation of CRT Reaction time across trials (40 trials per session)

- crt_commissions_mean: Mean of mean of CRT Commission errors across trials (40 trials per session)
- crt_commissions_sd: Standard deviation of mean of CRT Commission errors across trials (40 trials per session)

Based on studying the correlation plot only 2 (of the 6) complex reaction time variables were chosen as candidate explanatory variables: *crt_allRT_mean* (referred to as Complex reaction time (mean across sessions) Reaction time in Table 1 of the main article) and *crt_commissions_mean* (referred to as Commission errors in Table 1 of the main article).

Impulsivity (Item 12 and 17 from Barrat Impulsiveness scale, BIS-11) (2 variables)

- Impulsivity_mean: Mean of all impulsivity scores across sessions
- Impulsivity_sd: Standard deviation of all impulsivity scores across sessions

Based on the analysis of the correlation plot the *impulsivity_mean* (referred to as Impulsivity mean in Table 1 of the main article) is labelled as candidate explanatory variable.

SCL: Symptom Check List 5-item version (2 variables)

- SCL5TOT_mean: SCL-5 Total score Mean
- SCL5TOT_sd: SCL-5 Total score Sd

SCL5TOT_mean (referred to as Symptom Check List 5-item version in Table 1 of the main article) is chosen candidate explanatory variable.

Mobile sensors (2 variables)

- battery_use_mean: first the battery percent points used during a day is calculated and then this is averaged across sessions
- battery_use_sd: first the battery percent points used during a day is calculated and then the standard deviation this is calculated across sessions

The *battery_use_mean* (referred to as Mobile sensors: battery use (battery percentage points used during a day, mean across sessions)in Table 1 of the main article) is chosen as candidate explanatory variable.

In total 5 candidate explanatory variables are chosen. The strongest correlation within this set is between battery_use_mean and SCL5TOT_mean (-0.32).

For the logistic regression analysis (where the binary substance use episodes is the response) the craving mean is also included as a candidate explanatory variable, and we have strong correlation between the craving mean and SLC5TOT_mean (0.52), craving mean and battery_use_mean (-0.53) and craving mean and impulsivity_mean (0.44). The correlation between craving mean and the last two chosen candidate explanatory variables are -0.23 with crt_commissions_mean and -0.1 with crt_allRT_mean.
